# Individual and population level impacts of illicit drug use, sexual risk behaviours on sexually transmitted infections among young Aboriginal and Torres Strait Islander people: results from the GOANNA survey

**DOI:** 10.1186/s12889-016-3195-6

**Published:** 2016-07-19

**Authors:** Handan Wand, James Ward, Joanne Bryant, Dea Delaney-Thiele, Heather Worth, Marian Pitts, John M. Kaldor

**Affiliations:** Kirby Institute, University of New South Wales, Kensington, 2052 New South Wales Australia; South Australian Health and Medical Research Institute, Adelaide, South Australia Australia; Centre for Social Research in Health, University of New South Wales, Kensington, 2052 New South Wales Australia; Aboriginal Medical Service, Mount Druitt, Western Sydney Australia; School of Public Health and Community Medicine, University of New South Wales, Kensington, 2052 New South Wales Australia; Australian Research Centre in Sex, Health and Society, LaTrobe University, Melbourne, Victoria Australia

**Keywords:** Drug, Alcohol, Illicit drug use, Sexually transmitted infections, Population attributable risk, Aboriginal and Torres Strait Islander people, Australia

## Abstract

**Background:**

Sexually transmitted infections (STIs) have been increasing among Australian Indigenous young people for over two decades. Little is known about the association between alcohol and other drug use and sexual risk behaviours and diagnosis of STIs among this population.

**Methods:**

A cross-sectional, community based self-administered survey was conducted among young Aboriginal people aged 16–29 years of age. Questionnaires included socio-demographic characteristics, knowledge, sexual risk behaviours alcohol and other drug use and health service access including self-reported history of diagnosis with a STI. Logistic regression models and population attributable risks were used to assess individual and population level impacts of illicit drug use on high risk sexual behaviours and ever reported diagnosis of an STI.

**Results:**

Of the 2877 participants, 2320 (81 %) identified as sexually active and were included in this study. More than 50 % of the study population reported that they had used at least one illicit drug in past year. Cannabis, ecstasy and methamphetamines were the three most commonly used illicit drugs in the past year. The prevalence of self-reported STI diagnosis was 25 %. Compared with people who did not report using illicit drugs, risky alcohol use and sexual behaviours including inconsistent condom use, multiple sexual partners in the past year and sex with casual partners were all significantly higher among illicit drug users. In adjusted analysis, participants who reported using illicit drugs were significantly more likely to engage in sexual risk behaviours and to ever have been diagnosed with an STI. Adjusted Odds Ratios ranged from 1.86 to 3.00 (males) and from 1.43 to 2.46 (females). At the population level, more than 70 % of the STI diagnoses were attributed to illicit drug-use and sexual risk behaviours for males and females.

**Conclusion:**

Illicit drug use in this population is relatively high compared to other similar aged populations in Australia. Illicit drug use was associated with risky sexual behaviours and STI diagnoses among this study population. Developing and implementing effective STI prevention strategies should include not only safe sex messages but also include drug and alcohol harm reduction messages.

## Background

In Australia, young Aboriginal and Torres Strait Islander (hereafter Aboriginal) people are disproportionately represented in sexually transmissible infections (STI) notification data [1]. Representing just 3 % of the total population, yet accounting for 38 and 16 % of all gonorrhoea and chlamydia notifications in 2013, there remains an enormous gap between Aboriginal and non-Indigenous Australians. Further these rates have been disparate for over two decades, despite many program and policy interventions [1]. In addition to the high rates of STIs, previous studies have reported a relatively higher use of licit and illicit drugs such as alcohol, cannabis, ecstasy and methamphetamines among young Aboriginal people compared to non-Indigenous Australians [2]. Strong correlations between drug and alcohol use, and high risk sexual behaviours have been previously reported such as multiple/concurrent partners and infrequent condom use [3–5]. Methamphetamine has been previously reported to increase sexual pleasure and function and as such is associated with higher sexual risk behaviour and potential for STI including human immunodeficiency virus (HIV) transmission [6].

While various risk factors have been identified and associated with high risk sexual behaviours and STI diagnosis, their individual and population level contributions have not been investigated among Aboriginal people in Australia. In order to inform timely and effective STI prevention responses, we examined the dynamics of illicit drug and alcohol use and risky sexual behaviours. We particularly assessed their individual and population level contributions on STI diagnoses in Aboriginal people aged 16–29 who participated in a community based survey of sexual health and relationships.

## Methods

### Study design and sampling

A detailed description of the study population has been described elsewhere [7]. Briefly, the study population were Aboriginal women and men, aged 16–29 years, who were recruited at 40 Aboriginal one or two day cultural and sporting community events across Australia during the period of 2011 and 2013. Convenience sampling was used to recruit the study participants. The majority of events were mainly community events including several cultural celebrations (18/40), sports carnivals (15/40), annual regional show days/weekends (5/40) or Aboriginal community health events (2/40).

### Study population

The study collected demographic characteristics, sexual risk behaviour information including alcohol and other drug use, knowledge and self-reported diagnosis of STI from 2,877 individuals (59 % females and 41 % males). Of these, 2,320 (81 %) identified as being sexually active and are included in this analysis.

### Data collection

Questionnaires administered by peer workers on site. Consented participants completed the questionnaire using handheld personal digital assistants (PDAs). The PDAs were loaded with a specialised program to administer the prepared questionnaire and collect information in a de-identified, secure format. Participants were expected to read the questions and provide their responses by touching the screen with a small stylus. Audio recordings of the questionnaire were also available to the participants from the device. The software programs used for questionnaire design, survey administration, data collection and storage were Questionnaire Development System Design Studio (v2.6.1), HAPI (v2.6.1) and Warehouse Manager (v2.6.1) from NOVA Research Company. Current study included aboriginal men and women who were 16 to 29 years of age and consented to complete the survey. Overall, 8 % of the participants refused to complete the survey. Refusals were defined as not willing to complete the survey after survey collectors had explained the study to the participant. If an individual refused to complete the survey, no further information was collected and he/she was excluded from the study. However, Survey collectors recorded only the number refusals during set time periods at each event to gauge an understanding of the proportion of refusals overall for the study.

We focussed on factors previously identified as potential determinants of sexual risk behaviour including an STI diagnosis [7]: such as age (<20 years, 20–24 years, and 25+ years), level of completed education (< high school vs. high school or above), age at sexual debut (<16 years vs. 16+ years old), sexual identity (heterosexual vs. gay/bisexual); number of sexual partners in the past year (3 or more vs. <3); and being drunk/high (yes vs. no) at last sex as well as illicit drug use for the three most commonly used drugs in the previous 12 months, methamphetamine, ecstasy and cannabis. Other illicit drugs were grouped and included cocaine, heroin, petrol, fantasy, benzodiazepines, ketamine and acid were also collected as (yes/no). Each illicit drug used was categorized as a dichotomous measure. Condom use was measured using several questions including “How often did you use condoms in past year” (always vs. not always) and “Did you use a condom at last sex? (yes/no)”. Additional characteristics such as current smoker (yes vs. no) and alcohol intake status (7+ drinks/week vs. vs <7) were also considered as potential correlates of the primary outcome of interests. The primary outcome variables were dichotomous measures of (1) sexual risk behaviours (inconsistent condom use and/or three or more sexual partners in past 12 months) and (2) diagnosis with a STI (ever) among those who reported ever being tested for any STI.

### Statistical analysis

We used descriptive statistics (percentages) to characterise the study population according to their total number of illicit drugs used (more than once) in the last year. In this analysis, we first created a “*drug score*” for each individual by assigning a score of 1 (if illicit drugs were reported as being used) and 0 (otherwise); after we added the scores for each individual drug used, we re-categorized them according to their final *drug score*: (0: no drug, 1: one drug only, 2: two-drugs, 3: three-drugs and 4: four or more drugs).

We formally assessed the influence of socio-demographic characteristics; illicit drug use for the most commonly used illicit drugs, namely, cannabis, ecstasy and methamphetamine, and alcohol use on engaging in sexual risk behaviours and STI diagnosis separately.

For these analyses, sexual risk behaviour (yes vs. no) was defined as individuals who reported at least 3 or more sexual partners in the previous 12 months and not using condom in last sex. Age adjusted logistic regression models were used to determine whether illicit drug use was associated with sexual risk behaviours and a self-reported previous STI diagnosis. Adjusted odds ratios (aORs) and 95 % Confidence Intervals (CIs) are presented for women and men separately.

We tested the fitness of the model using the Hosmere-Lemeshow criteria [8]. The primary aim of this analysis was to assess whether the combination of risk factors under consideration could predict those at increased risk of an STI diagnosis with accuracy and an acceptable level of robustness.

We also calculated the population attributable risk (PAR) so as to estimate the proportion of sexual risk behaviours and STI diagnosis that could have been avoided if it was possible to modify some of the characteristics of the study population [9]. PAR is used as other standard statistical measures such as an odds ratio do not give sufficient evidence regarding their potential impact on disease occurrence. Briefly, PAR quantifies the population level impact of a risk factor of interest on the primary outcome of interest. The PAR is an epidemiological measure originally formulated as a function of odds ratio (OR) (from adjusted logistic regression) and the prevalence of risk factors of interest when there is only one risk factor at two levels (1 versus 0)

$$ PAR=\frac{p\left( OR-1\right)}{p\left( OR-1\right)+1}=1-\frac{1}{{\displaystyle {\sum}_{s=1}^2{p}_sO{R}_s}} $$

where *s* indexes the two strata determined by the value of a risk factor. We generalized the PAR into a multifactorial setting where there is more than one risk factor at multiple levels as follows:

$$ PAR=\frac{{\displaystyle {\sum}_{s=1}^S{p}_s\left(O{R}_s-1\right)}}{1+{\displaystyle {\sum}_{s=1}^S{p}_s\left(O{R}_s-1\right)}+1}=1-\frac{1}{{\displaystyle {\sum}_{s=1}^S{p}_sO{R}_s}} $$

where *OR*_*s*_ and *p*_*s*_, *s* = 1, …, *S*, are the odds ratios and the prevalence in the target population for the *s* being the combination of the risk factors [9].

The above equation can be interpreted as the proportion of the number of sexual risk behaviours and STI diagnosis that can be reduced if all known risk factors were eliminated or modified (at least theoretically) from the target population.

The PAR reflects the difference between the expected number of cases in the study population and the number of cases expected if all subsets of the members in the study population who were originally exposed to the modifiable risk factor(s) had eliminated their exposure(s) so that their odds ratios compared to the unexposed was 1, divided by the number of cases expected in the original cohort.

For this analysis, illicit drug use and risky alcohol use were all assumed to be modifiable risk factors. All of the PAR models were adjusted for age as it was considered to be a non-modifiable background risk factor. For the STI diagnosis outcome, sexual risk factors including inconsistent condom use and the number of recent sexual partners were also assumed to be modifiable risk factors. The PAR was estimated using adjusted odds ratios for each individual risk factor and their various combinations were calculated using logistic regression models. Prevalences for combinations of risk factors were estimated as multinomial probabilities among study participants at each unique level. Based on the methodology described above, gender-specific theoretical STI prevention strategies can be investigated.

Analyses were performed using Stata 12.0 (College Station, TX, USA) and SAS statistical software, version 9.3 (SAS Inc., Cary, NC, USA).

## Results

Of the 2877 participants who completed the survey, 2320 (81 %) of them were identified as sexually active (answered yes to ever had anal or vaginal sex) (40 and 60 % males and females respectively) and included in our analysis. Overall 42 % of this study population were aged between 16 and 20 years with over 50 % having less than a completed high school education. A total of 1029 (44 %) reported using illicit drugs in the previous 12 months. Of these, 869 (84 %) reported using cannabis; 344 (33 %) and 296 (29 %) reported using ecstasy and methamphetamine respectively in past year; 8 % (*n* = 83) of illicit drug users also reported injecting drug in past 12 months (data not shown). Cocaine use was reported by 11 % (*n* = 116) of illicit drug use participants and the least reported drugs were Lysergic acid diethylamide (LSD) (5 %), fantasy (4 %), benzodiazepines (2 %) and ketamine (2 %) (data not shown).

### Characteristics of the participants by patterns of drug-use categories

Table 1 presents the characteristics of the study participants across the 5-drug use categories (none, 1-drug, 2-drugs, 3-drugs and 4+ drugs) for women and men separately. There were no significant differences found by drug-use categories, by age group, nor sex. However there was an increasing trend between sexual risk behaviours and drug-use categories. Compared with women who did not use illicit drugs in the previous 12 months, those who reported using more drugs reported higher rates of not using a condom at last sex (64.2 vs. 71.1, 72.3, 81.8 and 81.1 %, for no-drug to 4+ drugs respectively). Similarly among men, an increasing trend of not using a condom at last sex was associated with higher poly drug use (55.9 vs. 65.4, 65.9, 80 and 72.7 %, for no-drug to 4+ drugs respectively). Higher proportions of people who used illicit drugs reported being high or drunk at last sex compared to those who did not use drugs [23.5 % (no-drug) vs. 31.4 % (1-drug) – 55.4 % (4+ drugs) for women and [24.4 % (no-drug) vs. 48.9 % (1-drug) to 68.2 % (4+ drugs)]. Among women who did not use illicit drugs in the previous 12 months compared with illicit drug users they were less likely to have had sex with someone they just met (casual partner) (6.9 vs. 8 % (1-drug) to 19.8 % (4+ drugs)), and similarly among men (14.2 vs. 18.7 % (1-drug) to 30.7 % (4+ drugs)). Self-reported STI diagnosis were more common among poly drug users compared to non-drug users (21.3 % (1-drug) - 42.6 % (4 + drugs) vs. 14.1 % (non-users) (women) and 27.7 % (1-drug) -52.4 % (4 + drugs) vs. 21.7 % (non-users).

**Table 1:** Characteristics of the sexually active study population (*n* = 2320) by poly-drugs (including methamphetamine, ecstasy, cannabis, cocaine, heroin, petrol, fantasy, benzo, ketamine, LSD-acid

	Males (*n* = 939)					Females (*n* = 1381)				
	No drug *N* = 479 (51 %)	1-drug *N* = 237 (24 %)	2-drugs *N* = 85 (9 %)	3-drugs *N* = 50 (5 %)	4+ drugs *N* = 88 (9 %)	No drug 812 (59 %)	1-drug 350 (25 %)	2-drugs 101 (7 %)	3-drugs 44 (3 %)	4+ drugs 74 (5 %)
Age groups										
16–19 years	219 (45.72)	95 (40.08)	32 (37.65)	18 (36.00)	31 (35.23)	287 (35.34)	120 (34.29)	34 (33.66)	14 (31.82)	21 (28.38)
20–24 years	157 (32.78)	75 (31.65)	30 (35.29)	15 (30.00)	31 (35.23)	264 (32.51)	128 (36.57)	35 (34.65)	17 (38.64)	31 (41.89)
25–29 years	103 (21.50)	67 (28.27)	23 (27.06)	17 (34.00)	26 (29.55)	261 (32.14)	102 (29.14)	32 (31.68)	13 (29.55)	22 (29.73)
Education										
<high school	236 (49.27)	144 (60.76)	51 (60.00)	24 (48.00)	54 (49.27)	236 (49.27)	144 (60.76)	51 (60.00)	24 (48.00)	54 (61.36)
Age at sex										
<16 years	372 (77.66)	191 (80.59)	69 (81.18)	41 (82.00)	68 (77.27)	502 (61.82)	248 (70.86)	76 (75.25)	36 (81.82)	58 (78.38)
Condom used in last sex										
No	268 (55.95)	155 (65.40)	56 (65.88)	40 (80.00)	64 (72.73)	521 (64.16)	249 (71.14)	73 (72.28)	36 (81.82)	60 (81.08)
Sexual identity										
Gay/Bisexual	18 (3.76)	18 (7.59)	7 (8.24)	4 (8.00)	20 (22.73)	16 (1.97)	13 (3.71)	5 (4.95)	4 (9.09)	3 (4.05)
Last person sex with										
Boyfriend/girlfriend	319 (66.60)	128 (54.01)	39 (45.88)	19 (38.00)	36 (40.91)	567 (69.83)	238 (68.00)	52 (51.49)	26 (59.09)	41 (55.41)
Someone just met	68 (14.20)	45 (18.66)	24 (28.24)	11 (22.00)	27 (30.68)	56 (6.90)	28 (8.00)	16 (15.84)	8 (18.18)	8 (10.81)
Other	92 (19.21)	64 (27.00)	22 (25.88)	20 (40.00)	25 (28.41)	189 (23.28)	84 (24.00)	33 (32.67)	10 (22.73)	15 (33.78)
Number of sexual partners										
3+	153 (31.94)	96 (40.51)	46 (54.12)	24 (48.00)	49 (55.68)	116 (14.29)	88 (25.14)	38 (37.62)	15 (34.09)	34 (45.95)
Current smoker										
Yes	122 (25.47)	140 (59.07)	52 (61.18)	31 (62.00)	65 (73.86)	265 (32.64)	216 (61.71)	64 (63.37)	33 (75.00)	56 (75.68)
Alcohol intake										
>7+ drinks/week	214 (44.68)	128 (54.01)	50 (58.82)	26 (52.00)	46 (52.27)	272 (33.50)	159 (45.43)	42 (41.58)	14 (31.82)	25 (33.78)
Drunk/high last sex										
Yes	117 (24.43)	116 (48.95)	40 (47.06)	28 (56.00)	60 (68.18)	191 (23.52)	110 (31.43)	50 (49.50)	20 (45.45)	41 (55.41)
Diagnosed with STI^a^										
Yes	37 (14.07)	33 (21.29)	18 (28.57)	15 (40.54)	23 (42.59)	126 (21.69)	77 (27.70)	26 (32.10)	14 (40.00)	22 (52.38)

^a^among tested

### Correlates of high risk sexual and drug use behaviours

Table 2 presents the frequency distribution and adjusted odds ratios for the risk factors stratified by sex. Older age groups (20–24 years and 25+ years), early age at sexual debut (<16 years old) and frequent alcohol intake (>7 drinks per week) were significantly associated with an increased prevalence of sexual risk behaviours in both women and men. Participants who used cannabis, ecstasy and methamphetamine in the previous 12 months also had significant correlates of sexual risk behaviours. Among women adjusted odds ratios for sexual risk behaviour among those who reported using cannabis were (aOR: 1.94, 95 % CI: 1.48, 2.55); for ecstasy (aOR: 2.03, 95 % CI: 1.30, 3.18) and methamphetamines (aOR: 2.14, 95 % CI: 1.32, 3.48); and for men: aOR for cannabis use was (aOR: 2.96, 95 % CI: 1.53, 3.05) for ecstasy (aOR: 3.00, 95 % CI: 1.53, 3.05) and methamphetamines (aOR: 2.25, 95 % CI: 1.33, 3.79). Smoking status was associated with increased levels of high risk sexual behaviours in males only; while less than a completed high school education appeared to have a significant impact on females only (aOR: 1.45, 95 % CI: 1.05, 2.00 and aOR: 1.38, 95 % CI: 1.08, 1.76 respectively).

**Table 2:** Factors associated with increased prevalence of high risk behaviours^a^ (condom not used in last sex and/or 3+ sexual partners in past 12 months): ^b^ age adjusted, among testers

	Males					Females				
	High risk sexual behaviors^a^ (*n* = 939)			STI diagnosis^b^ (*n* = 572)		High risk sexual behaviours (*n* = 1381)			STI diagnosis^b^ (*n* = 1017)	
	%	Adjusted OR^b^	*p*-value	Adjusted OR^b^	*p*-value	%	Adjusted OR^b^	*p*-value	Adjusted OR^b^	*p*-value
Age groups										
16–19 years	42 %	1		1		35 %	1		1	
20–24 years	33 %	1.74 (1.21,2.50)	0.003	1.12 (0.66,1.90)	0.680	34 %	1.68 (1.27,2.23)	<0.001	1.39 (0.96,2.01)	0.082
25+ years	25 %	2.06 (1.37, 3.11)	0.001	2.12 (1.27,3.52)	0.004	31 %	2.61 (1.91,3.56)	<0.001	1.60 (1.11,2.32)	0.012
Education										
< high school	46 %	1.08 (0.78,1.49)	0.637	1.18 (0.78,1.78)	0.444	49 %	1.38 (1.08,1.76)	0.011	1.40 (1.05,1.86)	0.022
Sexual identity										
Gay/Bisexual	7 %	1.67 (0.81,3.46)	0.165	2.73 (1.52,4.90)	0.001	3 %	1.26 (0.57,2.78)	0.570	1.02 (0.47,2.22)	0.968
Age at sexual debut										
<16 years old	79 %	1.89 (1.28,2.79)	0.001	0.98 (0.60,1.60)	0.922	67 %	2.00 (1.53, 2.58)	<0.001	1.60 (1.16,2.18)	0.004
Condom used last sex										
No	62 %	–	–	1.54 (0.98,2.40)	0.058	68 %	–	–	1.96 (1.39,2.77)	<0.001
Sex partners in past year										
3 or more	39 %	–	–	1.66 (1.11,2.49)	0.013	21 %	–	–	1.78 (1.29,2.45)	<0.001
Current smoker										
Yes	44 %	1.45 (1.05,2.00)	0.025	1.41 (0.94,2.11)	0.098	54 %	1.16 (0.91,1.49)	0.229	1.16 (0.88,1.54)	0.300
Alcohol intake										
>7+	49 %	1.85 (1.34,2.53)	<0.001	1.04 (0.69,1.55)	0.857	37 %	1.44 (1.11,1.87)	0.006	0.98 (0.74,1.31)	0.912
Drunk/high last sex										
Yes	63 %	2.39 (1.68, 3.40)	<0.001	2.27 (1.51,3.42)	<0.001	57 %	2.00 (1.48,2.65)	<0.001	1.74 (1.29,2.35)	<0.001
Methamphetamine used										
Yes	17 %	2.25 (1.33,3.79)	0.002	2.93 (1.84,4.67)	<0.001	10 %	2.14 (1.32,3.48)	0.002	1.80 (1.16,2.80)	0.009
Ecstasy used										
Yes	20 %	3.00 (1.79,5.03)	<0.001	2.98 (1.92,4.62)	<0.001	12 %	2.03 (1.30,3.18)	0.002	2.46 (1.63,3.70)	<0.001
Cannabis used										
Yes	40 %	2.16 (1.53,3.05)	<0.001	1.86 (1.24,2.79)	0.003	36 %	1.94 (1.48,2.55)	<0.001	1.43 (1.07,1.91)	<0.001

### Correlates of STI diagnoses

Being of an older age group, identifying as gay or bisexual, having three or more sexual partners in the past year and being drunk or high at last sex were all associated with a past STI diagnosis in men. In addition, cannabis, ecstasy and methamphetamine use were identified as significant correlates of increased prevalence of a self-reported STI diagnosis [(aOR: 1.86, 95 % CI: 1.24, 2.79); (aOR: 2.98, 95 % CI: 1.92, 4.62); (aOR: 2.93, 95 % CI: 1.84, 4.67)] respectively. Most of the risk factors identified as significant correlates for STI diagnosis for males were also identified among females. Particularly illicit drug use and being drunk or high at last sex showed strong associations with a self-reported previous STI diagnosis. Among women who reported using illicit drugs [(aOR: 1.94, 95 % CI: 1.48, 2.55); (aOR: 2.03, 95 % CI: 1.30, 3.18); (aOR: 2.00, 95 % CI: 1.48, 2.65) for cannabis, ecstasy and methamphetamine respectively were found. Early sexual debut (<16 years) was found to be a significant predictor of a previous STI diagnosis among females only.

### Population level impacts of risk factors on high risk behaviours

In sex-specific analysis (Table 3), cannabis use was associated with the highest PAR compared to ecstasy and methamphetamines in both women and men; 32 % (95 % CI: 25, 39 %) and 24 % (95 % CI: 20, 29 %) of the high risk sexual behaviours were attributed to cannabis use for men and women respectively. Relatively high prevalence and odds ratios were responsible for this high impact [36 and 40 % (prevalences); 1.94 and 2.16 (odds ratios) for women and men respectively]. Approximately one third of sexual risk behaviours were associated with alcohol intake (>7 drinks/week) among males; being drunk or high at last sex was associated with the highest PAR with 34 % among males; while 21 % of the high risk behaviours were associated with this factor among females.

**Table 3:** Population level impact of (potentially) modifiable risk factors

Potentially modifying risk factors	High risk sexual behaviours^a^		STI Diagnosis^b^	
	PAR% (95 % CI)		PAR% (95 % CI)	
Drug & alcohol related	Male	Female	Male	Female
Cannabis use	32 % (25, 39 %)	24 % (20, 29 %)	27 % (20, 36 %)	13 % (10, 17 %)
Ecstasy use	29 % (22, 37 %)	11 % (9, 14 %)	30 % (25 37 %)	13 % (10, 16 %)
Methamphetamine use	20 % (14, 25 %)	11 % (8, 14 %)	28 % (22, 34 %)	7 % (5, 10 %)
Alcohol intake (7+/week)	30 % (23, 36 %)	14 % (11, 18 %)	–	–
Drunk/high last sex	34 % (28, 40 %)	21 (17, 25 %)	31 % (24, 39 %)	17 % (13, 21 %)
High risk sexual behaviours^a^				
No condom use in last sex	–	–	72 % (62, 80 %)	40 % (30, 49 %)
+3 or more sexual partners^d^				
Modifying drug use				
Cannabis use				
+ Ecstasy use	45 % (36, 51 %)	30 % (24, 35 %)	43 % (34, 51 %)	20 % (15, 25 %)
+ Methamphetamine use				
Modifying drug/alcohol				
+ high risk sexual behaviours^†^				
All drug related				
+ Alcohol related^c^	76 % (71, 80 %)	70 % (65, 73 %)	74 % (66, 80 %)	70 % (63, 75 %)
+ No condom use last sex				
+3 or more sexual partners^d^				

^a^not used condom in last sex, and/or 3+ sexual partners past 12 months; ^b^among testers

^c^including only drunk/high in last sex for the “STI diagnosis”; ^d^past 12 months

The combined impact of all drug related factors, namely cannabis, ecstasy, cocaine, associated with 45 % (95 % CI: 36, 51 %) and 30 % (95 % CI: 24, 35 %) of the high risk sexual behaviours among men and women respectively. When these three-drug related factors were combined with the alcohol related factors, PAR increased to 76 % (95 % CI: 71, 80 %) and 70 % (95 % CI: 65, 73 %) for females and males respectively (Table 3).

### Population level impacts of risk factors on STI diagnosis

Individual and combined level impacts of drug, alcohol and high risk sexual behaviour factors on self-reported STI diagnosis are presented in Table 3 for men and women separately. High risk sexual behaviours, namely, not using a condom at last sex and having 3 or more sexual partners in the past year were considered together and appeared to have the largest impact on STI diagnosis in males and females (PAR: 72, 95 % CI: 62, 80 % and PAR: 40, 95 % CI: 30, 49 % for males and females).

Among men, the population level impacts of cannabis, ecstasy, methamphetamine use on STI diagnosis were estimated to be 27, 30 and 28 % respectively. When all these drugs were combined, PAR increased to 43 % (95 % CI: 34, 51 %); while these drugs were collectively responsible for 20 % (95 % CI: 15 %, 25 %) of all the diagnoses among females. Finally, our data suggested that 74 % (95 % CI: 66 %, 80 %) of the all the STI diagnoses were attributed to the seven modifiable risk factors among males. This proportion was 70 % (95 % CI: 63, 75 %) among females. Goodness of fit measures of the adjusted models were ranged from 0.091–0.989 (for males) and 0.423–0.612 (for females) for both primary outcome variables.

## Discussion

To our knowledge, this is the first study to report individual and population level impacts of combinations of risk factors including alcohol and illicit drug use on sexual risk behaviours and their associations with a past STI diagnosis among young Aboriginal people. This study highlights significant interplay between illicit drug use, sexual risk behaviours and self-reported past STI diagnosis among this population with the finding that the majority of risky sexual behaviours and past STI diagnosis were associated to drug and alcohol use in both women and men. The three most commonly used illicit drugs by this study population, (cannabis, ecstasy and methamphetamines), collectively accounted for 45 and 30 % of the high risk behaviours among males and females respectively. Together alcohol and illicit drugs showed the strongest associations with risky sexual behaviours. This suggests that reducing alcohol and illicit drug use would make a significant impact on STI transmission in this population.

Assessing the impact of individual risk factors for STIs has important implications for prevention policy as well as clinical practice. Previous studies have shown the link between STI diagnosis, and sexual risk behaviour and our study concurs with these findings [3, 10–14]. Currently in Australia the two professional fields of health prevention of alcohol and other drugs and sexual health are often delivered separately and rarely is there cross over in the workforce; however our data show many reasons why these two fields should be merged or work more closely with each other. Models of care should be explored such as testing for STIs, including HIV as well as other blood borne viruses such as hepatitis B and C. Further characterizing, identifying and targeting individuals with both alcohol and other drug use issues and who engage in sexual health risk behaviour will most likely play a significant role in the trajectory of diagnosis with sexually transmitted infections. It is now accepted that illicit drug users routinely engage in high-risk sexual behaviours that put them at an increased risk of contracting sexually transmitted infections including HIV [15, 16].

Our study also shows the impact that alcohol and other drug use has at a population level on sexual risk behaviours. In our study population, relatively risky alcohol misuse was found to be associated with risky sexual behaviours at a population level (PAR%: 50 and 40 % for males and females respectively) as well as having strong associations with a past STI diagnosis (aOR: 1.68 and 1.69 for males and females respectively). While this field has been rarely investigated in Australia among young Aboriginal people much more work is required to assess the impact of alcohol restrictions on STI and sexual risk behaviour. A study of alcohol restrictions in the Kimberley region witnessed a decrease in STI notifications [17]. Much more work is required to assess this impact.

Our findings also indicate that more than 80 % of the self-reported STI diagnoses were associated with seven modifiable risk factors among males; while 60 % of the self-reported STI diagnoses were attributed to these modifiable factors when the study population is restricted to females only. The difference between the males and females were due to the differences in the prevalences of the drug/alcohol related factors; particularly methamphetamine, ecstasy use, frequent alcohol intake and being drunk at last sex were more common among males compared to the females. In terms of high risk sexual behaviours, lack of condom use in last sex was slightly higher among females (68 vs. 62 %) while having higher number of sexual partners past 12 months almost doubled among males compared to females (39 vs. 21 %). Consistent with these estimates, more than 70 % of the self-reported STI diagnoses were attributed to the high risk sexual behaviours (namely lack of condom use in last sex and higher number of sexual partners) among males compared to 40 % among females.

The PAR findings for the self-reported past STI diagnoses suggest that among males almost three quarters of these diagnoses (72 %) could be avoided by reducing the high risk sexual behaviours (i.e. increasing condom use and decreasing number of sexual partners); while these factors collectively accounted for 40 % of the diagnoses among the females.

This study has several limitations; therefore the results should be interpreted accordingly. First the representativeness of participants recruited from community events may be questionable as those who attend these events may have different characteristics than those who do not; particularly the way that they are connected to their communities, their knowledge regarding sexually transmitted infections, and use of health services compared to other young Aboriginal individuals. Nevertheless, we are not aware of another means of recruiting a large, diverse young Aboriginal people as strategies based on household or telephone recruiting may potentially under-represent Aboriginal individuals. However the proportion of participants who self-reported ever having an STI diagnosis is consistent with available epidemiological evidence on STI positivity in the population [1].

Current study was designed as a cross-sectional survey; therefore it shares the same limitations of these types of study designs such as lack of temporal associations between exposure and outcomes of interest. Testing, diagnosis and behavioural data were all collected by self-report and may be subject to both recall bias and measurement error. The main limitation of the study however is the lack of biological testing results for previous STI diagnoses. However, we did find strong associations between participants who used illicit drugs and alcohol with risky sexual behaviours compared to those who did not use alcohol and illicit drugs. Similarly we found strong association between those who used alcohol and illicit drugs and a previous self-reported STI diagnoses compared to those who had not used alcohol at risky levels or illicit drugs. Therefore, we conducted the analyses in two-stages: first we identified the factors associated with risky sexual behaviours (e.g. combination of lack of condom use and higher number of sexual partners); and secondly, we assessed the associations between the high risk behaviours along with other socio-demographic factors with a past self-reported STI diagnoses. Our results indicate that the majority of the factors associated with high risk sexual behaviours were also associated with high prevalence of past STI diagnosis.

There is an urgent need to focus on new STI prevention intervention strategies that will not only address the safe sex behaviour of young Aboriginal people, but also encourage reducing illicit drug use and risky alcohol intake.

## Conclusion

The impact of risk factors for a disease at a population level has important implications for prevention policy and practice. In our study we have shown that the majority of the high risk sexual behaviours and STI diagnoses were associated to using illicit drug use and risky alcohol use. The PAR findings suggest that the largest number of STIs could be avoided by reducing high risk sexual behaviours as well as drug and alcohol intake.

## Abbreviations

Aboriginal, Aboriginal and Torres Strait Islander; aOR, Adjusted odds ratio; PAR, population attributable risk; PDA, personal digital assistants; STI, sexually transmitted infections
